# Does Supersonic Shear Wave Elastography Help Differentiate Biliary Atresia from Other Causes of Cholestatic Hepatitis in Infants Less than 90 Days Old? Compared with Grey-Scale US

**DOI:** 10.1155/2019/9036362

**Published:** 2019-06-02

**Authors:** Xingxing Duan, Ya Peng, Wengang Liu, Liu Yang, Jie Zhang

**Affiliations:** ^1^Department of Ultrasound, Hunan Children's Hospital, Changsha, Hunan Province 410007, China; ^2^Department of Ultrasound, The Third Xiangya Hospital of Central South University, Changsha, Hunan Province 410013, China

## Abstract

**Purpose:**

To investigate the diagnostic performance of shear wave elastography (SWE) for measuring liver stiffness to identify and differentiate biliary atresia (BA) from cholestatic hepatitis in infants younger than 90 days.

**Methods:**

A total of 138 infants younger than 90 days with cholestatic hepatitis were examined by SWE. The infants were subclassified into BA and nonbiliary atresia (non-BA) groups. Receiver operating characteristic (ROC) analysis was used to determine the sensitivity and specificity of hepatic Young's modulus measurements, the ultrasonic findings in the differential diagnosis of suspected BA, and the cut-off value to diagnose BA.

**Results:**

In all infants with cholestatic hepatitis, the cut-off value of hepatic Young's modulus to differentiate the BA group from the non-BA group was 12.35 kPa and the area under the ROC curve (AUC) was 0.937, with a sensitivity of 84.3% and a specificity of 89.7%; nevertheless the AUC of the abnormal gallbladder (AbGB) was 0.940, with a sensitivity of 96.1% and a specificity of 92.0%. In the parallel test, triangular cord (TC) sign combined with AbGB had the best diagnostic performance and the AUC was 0.960, with a sensitivity of 100% and a specificity of 92.0%. In the serial test, SWE combined with AbGB achieved the best diagnostic performance; the AUC was 0.902, the sensitivity and specificity were 80.4% and 100%, respectively.

**Conclusions:**

SWE could not only help differentiate BA from cholestatic hepatic diseases but also increase the diagnostic specificity when combined with grey-scale ultrasound in the serial test.

## 1. Introduction

Biliary atresia (BA) is a pediatric hepatic disease of unknown etiology that is characterized by biliary obliteration and progressive liver fibrosis [1]. BA incidence varies among different countries of the world and is higher in Asia than in Europe and North America [2, 3]. The clinical manifestations include progressive jaundice, deep-colored urine, light-colored stools, and an increase in total serum bilirubin, especially direct bilirubin. The similarity in clinical presentation and blood biochemistry results between patients with BA and those with infantile hepatitis syndrome represents a great challenge to clinicians in establishing a final diagnosis [4]. However, the treatment of the two diseases is very different, with BA requiring surgery and infantile hepatitis often cured with only a medical treatment [5, 6]; e.g., reduced glutathione protects the liver, ursodeoxycholic acid promotes bilirubin excretion, and bifendate reduces aminotransferase. The Kasai procedure is the first-line treatment for BA [7], although there is a controversy regarding the best operation time for biliary drainage [5, 8, 9]. It is widely accepted that patients should undergo surgery within 90 days of birth and ideally within 60 days to achieve optimal results [10, 11]. Thus, a prompt diagnosis is essential for a correct treatment decision [12].

At present, the ultrasound-guided percutaneous liver biopsy is regarded as the most effective method and the gold standard for preoperative BA diagnosis [13]. There are deficiencies in this approach that should be not ignored. In addition to being invasive, a deep sedation is required for infants and there are risks of hemorrhage and bile leakage. Furthermore, it is not able to assess the stiffness of the whole liver because of the small size of the biopsy specimen [14]. A newly developed percutaneous ultrasound-guided cholecystocholangiography with micro bubbles is considered to be an effective approach to diagnose BA. However, it is also invasive, needs anesthesia, and presents risks of hemorrhage and bile leakage [15]. Furthermore, micro bubble contrast agents such as SonoVue have not yet been officially approved for pediatric use in China.

Ultrasonography is the most common noninvasive examination method used for BA investigation [16]. BA ultrasound features include triangular cord (TC) sign, gallbladder abnormalities (e.g., shape, decreased contractility, and pseudogallbladder), invisible common bile duct, hepatic artery abnormalities (e.g. dilatation, increase in velocity), visible hepatic subcapsular color Doppler flow signal, porta hepatis microcyst, and an enlarged liver or an abnormal liver echo [17–23]. However, BA diagnosis can be extremely difficult early in the disease course or where there are atypical ultrasonic images such as invisibility of the TC sign, irregular gallbladder shape, or uniform hepatic echo texture on grayscale sonography [24]. In these cases, the application of SWE to measure hepatic stiffness appears as a new and effective technique for BA early diagnosis and differential diagnosis.

In this study, we performed SWE to obtain Young's modulus value on infants less than 90 days old in whom cholestatic hepatitis was suspected clinically, in combination with grey-scale ultrasonic findings, with the aim to assess SWE diagnostic performance in identifying and differentiate BA from cholestatic hepatitis in infants younger than 90 days by comparing this approach with grey-scale ultrasonography.

## 2. Materials and Methods

The study was approved by the Ethics Committee of Hunan Children's Hospital (ChangSha City, Hunan Province, China, Number: HCHLL-2017-03). Informed written consent was obtained from each of the participant's parents and was conducted between 1 November 2016 and 1 December 2017.

### 2.1. Patients

A total of 138 infants (76 males and 62 females, 5-90 days old) clinically suspected of having cholestatic hepatitis were included in our study. These infants were further classified into BA and non-BA groups according to their operative and postoperative pathological findings, or liver biopsy, blood biochemistry examination, and clinical assessment after medical treatment. An automatic biochemical analyzer (AU5800, Beckman Coulter, America) was used for measurements; approximately 2 ml of venous blood from each infant was collected after fasting 4 hours within three days before or after SWE examination. Measurements of total bilirubin (TBIL), direct bilirubin (DBIL), indirect bilirubin (IBIL), and *γ*-Glutamyl transpeptidase (*γ*-GT) were based on Enzyme reaction rate method. Additional 62 infants (28 males and 34 females, 7-90 days old) were enrolled as a healthy control group. Inclusion criteria for healthy controls were born at term (37-42 weeks), liver function found no abnormal changes (TBIL: 3.4-17.0*μ*mol/L, DBIL≤6.0*μ*mol/L, IBIL: 3.0-17.0*μ*mol/L, *γ*-GT≤32.0IU/L, aspartate aminotransferase≤40.0IU/L, alanine aminotransferase≤40.0IU/L, total bile acid≤9.67*μ*mol/L), normal abdominal imaging, and no evidence of jaundice, intrauterine infection, pneumonia, acute respiratory distress syndrome, asphyxia, hereditary metabolic disorders or congenital heart diseases. Exclusion criteria were: prematurity, small for gestational age (<37 weeks), and the presence of macrosomia, abnormal liver function, increased hemobilirubin, viral hepatitis, congenital heart disease, congenital hepatic fibrosis, congenital cholangiectasis, polycystic kidney, or abnormal abdominal imaging. All participants underwent hepatobiliary ultrasonography under fasting conditions (>4 hours), with SWE applied to obtain Young's modulus value.

### 2.2. Ultrasound and SWE Scan Technique

The ultrasound and SWE examinations were performed by a radiologist with more than 10 years' experience in abdominal sonography and 3 years' experience in elastography on a TUS-Aplio 500 scanner (Toshiba Medical Systems, Tokyo, Japan). All scans were performed using a 14L5 linear array probe (10 MHz).

The grey-scale ultrasonography examination was performed after placing the infants in the supine position and the 14L5 linear probe was placed on the abdomen to carefully scan the liver, gall bladder, and spleen. Infants should fast at least for 4 hours and the gall bladder size was measured 2 times: the first time was before the meal and the second time was one hour after the last feed. Then the contractibility of the gall bladder was calculated. The existence of these grey scale ultrasonic signs should be carefully identified as follows: (1) the triangular cord (TC) sign: a triangular or layer of high echo in front of the portal vein, with a thickness≥3 mm; (2) abnormal gallbladder (AbGB): stiff, small or irregular gallbladder or absence of gall bladder; the gallbladder contractibility<50% was considered to be abnormal.

SWE measurements of liver stiffness were obtained while the infants were breathing quietly. The infants lay supine in a resting state and the examination was performed by placing the transducer probe vertically just below the right costal margin. The rectangular sample frame was approximately 15 × 15 mm in size and was placed 10 mm below the hepatic capsule of the lower segment of the right anterior hepatic lobe. A circular region of interest (ROI) of approximately 8 to 10 mm in diameter was then drawn in the center of the sample frame, avoiding hepatic vessels and the gallbladder. The display modes for SWE including speed, elasticity, and propagation modes were obtained and could change when images were frozen. The elastic modulus was obtained in the form of an SWE map in kPa in the elasticity mode and the arrival time contour through the propagation mode. Regular and straight contour lines indicated that elastic waves propagated as expected and the reliability of the data was high. Conversely, bent and irregular contour lines suggested low data reliability and that another SWE measurement was required to achieve a reasonable result. Moreover, the wider the interval between the contour lines, the harder the tissue. Five elastography images were obtained for each participant in accordance with the following quality control specifications: the arrival time contour was regular and straight with a standard deviation (SD) less than 10% of the average value. Young's modulus value of the elastography images was calculated and the mean values were included in the statistical analysis.

### 2.3. Statistical Analysis

Statistical analysis was performed using SPSS version 18.0 software (IBM Corporation, Armonk, NY). Data were reported as mean ± SD. Multiple comparisons among multiple samples were performed by one-way ANOVA and least significant difference (LSD)-*t* test. The correlation between Young's modulus value and age, TBIL, DBIL, IBIL, and *γ*-GT in the patients with BA were analyzed by Spearman correlation and linear regression. The AUC of the typical ultrasound findings, Young's modulus value, and their combination to diagnose BA were calculated with ROC. The cut-off value of Young's modulus in identifying BA was obtained according to Youden Index, which was also used to evaluate the sensitivity and specificity. In order to find the largest number of infants who were suspected of having BA, we used the parallel test; that is to say, if any one of the observation indexes was positive, the test was considered as positive, to improve the diagnostic sensitivity. When ultrasonic findings were atypical, to reduce the economic and psychological burden of non-BA patients and reduce unnecessary invasive operations, we used the serial test; that is to say, if all of the observed indexes were positive, the test was considered as positive; otherwise, if at least one of the observed indexes was negative, the test was considered as negative, to improve the diagnostic specificity.* P* <0.05 was considered statistically significant.

To assess the SWE intraobserver error, 40 subjects were randomly recruited. SWE was performed by the same radiologist before and after two-dimensional gray-scale ultrasonography examination and in both before and after examination the SWE value was measured 5 times. The mean was calculated for further statistical analysis. Intraclass correlation coefficient (ICC) and 95% confidence intervals (95% CI) were calculated; ICC values greater than 0.75 indicated excellent reliability.

## 3. Results

### 3.1. Patient Characteristics

Among the 138 infants suspected of having cholestatic hepatitis, 51 were diagnosed as having BA via surgery and pathological findings. The remaining 87 infants had a condition other than BA (e.g., neonatal hepatitis, infantile hepatitis syndrome, cytomegalovirus infection, or Citrin protein deficiency syndrome) diagnosed by surgery or liver biopsy and/or blood biochemistry examination and clinical assessment and for this reason they were assigned into the non-BA group. Gender and age characteristics of the three groups are shown in Table 1.

### 3.2. Grey Scale Ultrasonic Findings

Among the 51 BA patients, 14 were without TC sign, including 13 within 30 days of age and one with 49 days of age. The TC sign was negative in all non-BA patients. Among all BA patients, only in 2 the typical gallbladder abnormalities were absent, while 7 of the 87 non-BA patients had an abnormal gall bladder.

### 3.3. Intraobserver Diversity

An excellent reliability was found within repeated measurements by the same radiologist, with an ICC of 0.873 and 95% CI from 0.772 to 0.931.

### 3.4. Comparison of Young's Modulus Values between Different Groups within the Same Gender and between Genders within the Same Group

**Figure 1:**
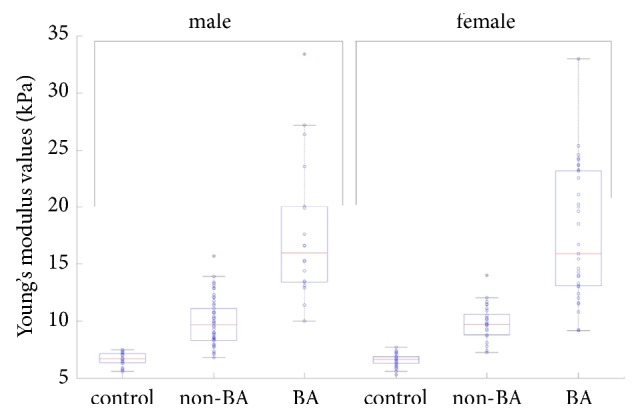
Box-and-whisker plot showing the distribution of liver stiffness values in control, non-BA and BA group when stratified by gender. Circle with a red cross in plots indicates the maximum stiffness value.

No statistically significant difference was observed in Young's modulus values between genders within the same group (*P*>0.05). However, the values varied significantly between different groups within the same gender (*P*< 0.01), as shown in Table 2 and Figure 1.

### 3.5. Comparison of Young's Modulus Values among Different Groups

**Figure 2:**
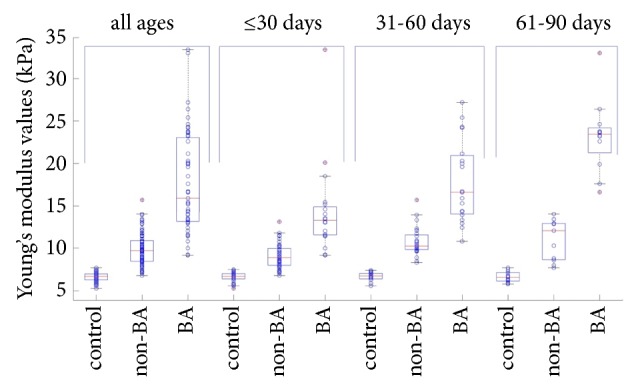
Box-and-whisker plot showing the distribution of liver stiffness values in control, non-BA and BA group when stratified by all ages, ≤30 days, 31-60 days, and 61-90 days. Circle with a red cross in plots indicates the maximum stiffness value.

**Figure 3:**
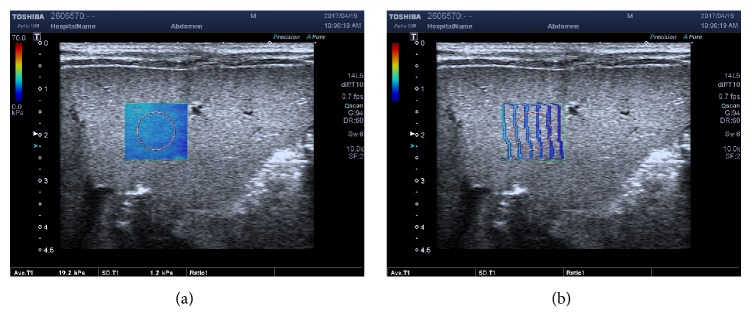
Biliary atresia in a 45-days-old boy. (a) SWE map in the elasticity mode; Young's modulus value of ROI was 19.2 kPa, SD=1.2 kPa. (b) SWE map in the propagation mode; the arrival time contour of the same case was regular and the interval between the contour lines was wider.

Young's modulus values varied significantly between groups (*P* < 0.01) and ranged from 5.30 to 7.70 kPa, 6.80 to 15.70 kPa, and 9.17 to 33.40 kPa in the control, non-BA, and BA groups, respectively. Moreover, Young's modulus values differed with age in both non-BA and BA group (*P* <0.01), as shown in Table 3 and Figures 2 and 3.

### 3.6. Relationship between Young's Modulus Values (kPa) and Age, TBIL, DBIL, IBIL, and *γ*-GT in the BA Group (n=51)

**Figure 4:**
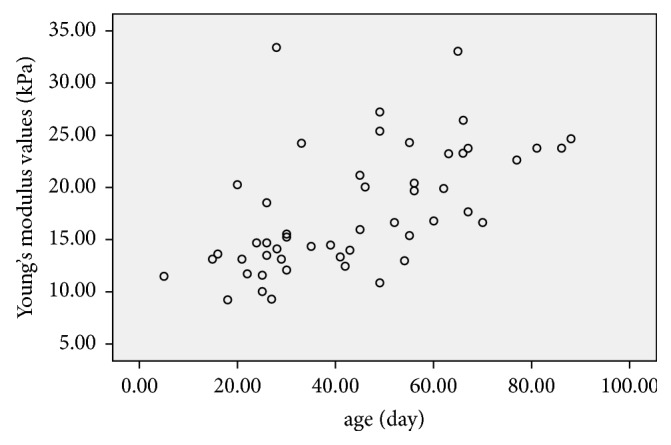
Scatter plot of the correlation between Young's modulus values (kPa) and age (day) in the BA group, the correlation coefficient was 0.642,* P*<0.001.

**Figure 5:**
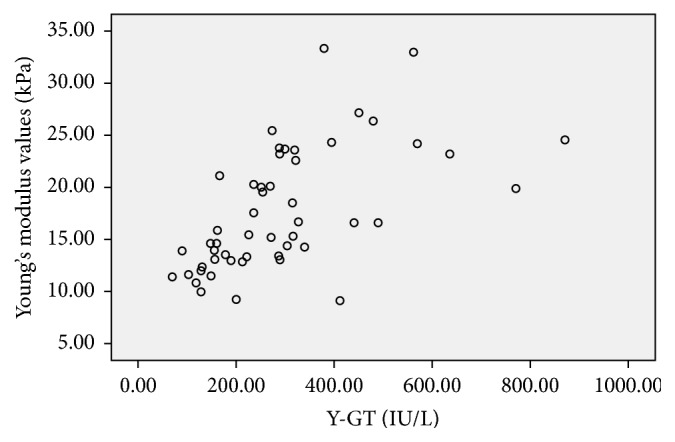
Scatter plot of the correlation between Young's modulus values (kPa) and *γ*-GT (IU/L) in the BA group, the correlation coefficient was 0.678,* P*<0.001.

**Figure 6:**
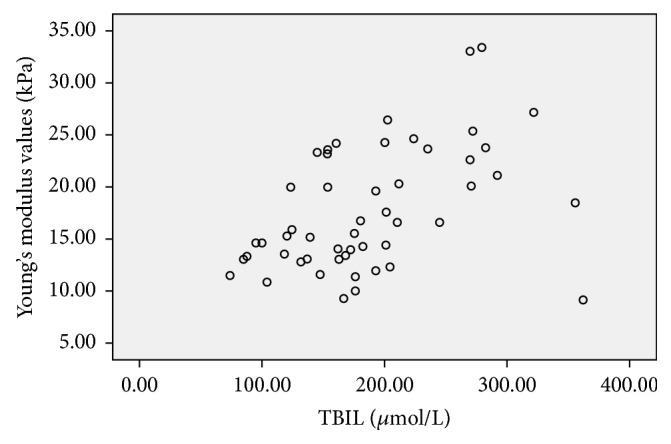
Scatter plot of the correlation between Young's modulus values (kPa) and TBIL (*μ*mol/L) in the BA group, the correlation coefficient was 0.467,* P* value was 0.001.

**Figure 7:**
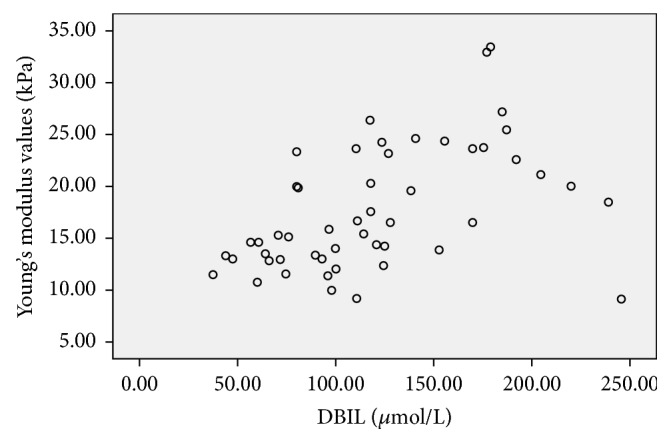
Scatter plot of the correlation between Young's modulus values (kPa) and DBIL (*μ*mol/L) in the BA group, the correlation coefficient was 0.548,* P*<0.001.

**Figure 8:**
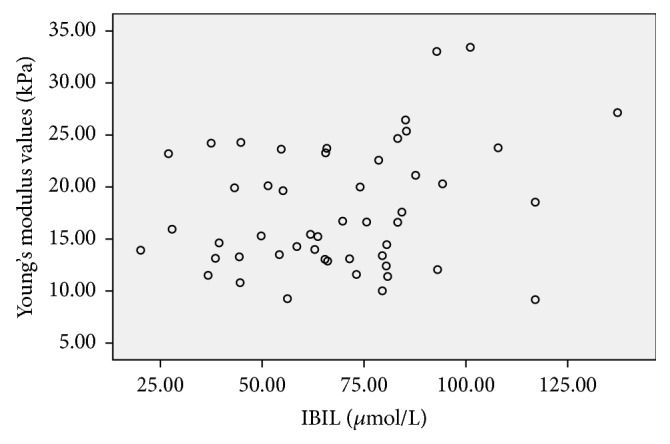
Scatter plot of the correlation between Young's modulus values (kPa) and IBIL (*μ*mol/L) in the BA group, the correlation coefficient was 0.222, and* P *value was 0.117.

All the blood biochemical tests were performed within 3 days of SWE examination. A correlation analysis showed that age, TBIL, DBIL, and *γ*-GT levels were all significantly correlated with liver Young's modulus value (kPa) (all *P* value<0.01), whereas IBIL did not significantly correlate with liver Young's modulus value (*P*>0.05), as shown in Table 4 and Figures 4–8.

A linear regression analysis showed that age (*P*=0.012) and *γ*-GT (*P*=0.014) significantly correlated with hepatic Young's modulus value (kPa), whereas TBIL and DBIL did not. Furthermore, age had a greater effect on the above results than *γ*-GT, as shown in Table 5.

### 3.7. Diagnostic Performance of Hepatic Young's Modulus Value from SWE, Typical Ultrasound Findings, and Their Combination to Diagnose BA in All Infants with Cholestatic Hepatitis

**Figure 9:**
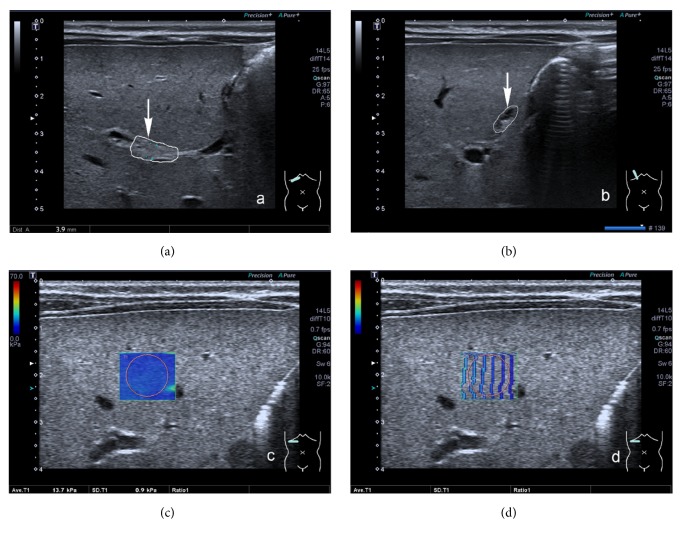
Biliary atresia in a 60-days-old girl. (a) The arrow points to the triangular cord sign and its thickness was 3.9 mm. (b) The arrow points to the abnormal gall bladder, small in size, and irregularly shaped. (c) The SWE image shows Young's modulus value of 13.7 kPa and SD of 0.9 kPa. (d) The quality control SWE images show that the shear wave propagation curve was regular.

**Figure 10:**
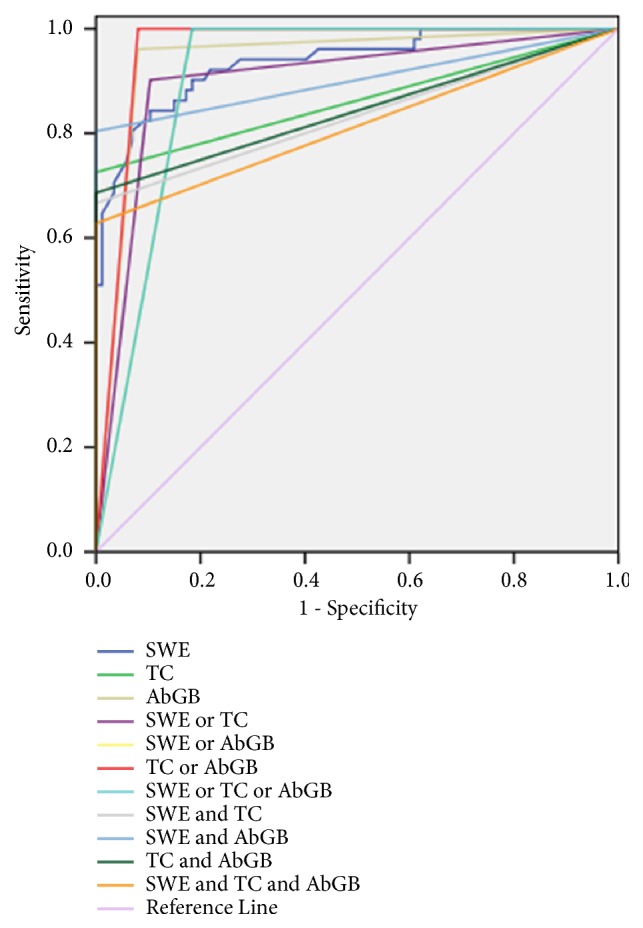
ROC of SWE, typical grey-scale ultrasound findings, and their combination to diagnose biliary atresia in all infants with cholestatic hepatitis (AbGB: abnormal gall bladder, SWE: shear wave elastography, TC: triangular cord sign, or: in the parallel test, and: in the serial test).

The AUC of Young's modulus value to diagnose BA in patients who were clinically suspected of having cholestatic hepatitis was 0.937. The best cut-off value was 12.35 kPa, with a sensitivity of 84.3% and a specificity of 89.7%. The combination of TC sign and AbGB in the parallel test resulted in the best diagnostic performance, with an AUC of 0.960, while the combination of SWE and AbGB in the serial test resulted in the best diagnostic performance, with an AUC of 0.902, as shown in Table 6 and Figures 9 and 10.

### 3.8. Diagnostic Performance of Hepatic Young's Modulus Values from SWE, Abnormal Gallbladder, and Their Combination to Diagnose BA in Patients without TC Sign

**Figure 11:**
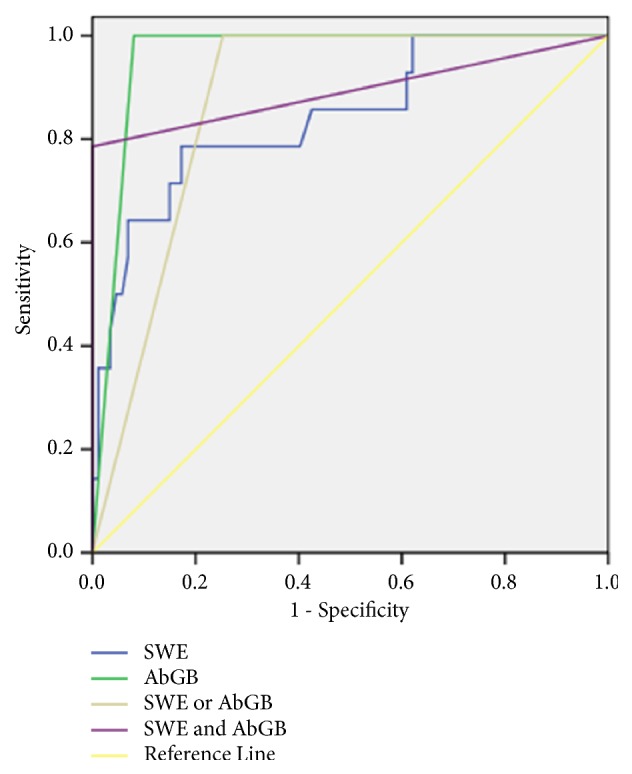
ROC of hepatic Young's modulus values of SWE, grey-scale ultrasound finding, and their combination to diagnose biliary atresia in patients without TC sign. (AbGB: abnormal gall bladder, SWE: shear wave elastography, or: in the parallel test, and: in the serial test).

In patients without TC sign, the AbGB was more useful to diagnose BA and the AUC was 0.960, with a sensitivity of 100% and a specificity of 92%. The cut-off value of hepatic Young's modulus to diagnose BA was 11.55 kPa and the AUC was 0.842, with a sensitivity of 78.6% and a specificity of 82.8%. If the combination of SWE measurement and the AbGB was used in the serial test to diagnose BA, the specificity raised to 100%, as shown in Table 7 and Figure 11.

## 4. Discussion

The development of elastography ultrasound techniques led to widespread and standard application in hepatic diseases. Although few reports are available describing the use of SWE in the diagnosis and differential diagnosis of BA, the comparability between them is hardly possible because of the ultrasound systems and their elastography imaging mode that are not completely the same [6, 25, 26].

In this study, we used the SWE developed by Toshiba Aplio 500. Few reports are available regarding the use of Aplio 500 for liver elastography in adults and infants [27–29]. In this article, a high correlation within repeated measurements was found by the same radiologist, with an ICC of 0.873 and 95% CI from 0.772 to 0.931, which indicated that the SWE of this ultrasound system was also suitable for infants and newborns' liver.

On the basis of our results, these values were higher in the BA and non-BA groups than in the control group, and the mean of Young's modulus values in BA group was significantly higher than in the non-BA group. This was likely the result of the differences in the disease process, with BA involving destruction and proliferation of the intrahepatic bile duct, cholestasis of hepatic cells and bile capillaries, and progressive portal fibrosis [30].

In our study, we found that Young's modulus values were higher in the BA group than in the non-BA group in patients within the same age group. As regards the BA group, the values were higher in the 31 to 60 days of age group than in the ≤30 days of age group, and in the 61 to 90 days of age group than in the 31 to 60 days group. The correlation analysis results showed that Young's modulus values positively correlated with age in patients with BA, and this was consistent with other studies [6, 26]. This is because as the patients got older, the degree of hepatic fibrosis got more severe, so as the stiffness of the liver. Hepatic Young's modulus values had a positive correlation with level of TBIL, DBIL, and *γ*-GT in blood serum, indicating that cholestasis and inflammation of bile duct might affect hepatic stiffness.

The best cut-off value in the ROC curve to diagnose BA in all suspected patients was 12.35 kPa, with a sensitivity of 84.3%, a specificity of 89.7%, and an area under the ROC curve of 0.937. This indicated that SWE was a useful tool for the early differential diagnosis of BA, as previously observed [13, 26]. Wang et al. [6] used an AixPlorer ultrasound system to examine 38 infants aged 16 days to 5 months with BA. Their results showed that hepatic Young's modulus in the BA group was 20.46±10.19 kPa, with a cut-off value of 8.68 kPa differentiating between BA and control groups. In their work, the area under the ROC curve was 0.997, and the sensitivity and specificity were 97.4% and 100%, respectively. The results of our study were not entirely consistent with those of Wang et al. for several reasons. First, we examined the efficiency of Young's modulus in diagnosing BA in all suspected cases, not in the healthy control group and BA group. Second, different ultrasound systems and calculation methods would affect their SWE measure results [31, 32]. Third, the sample size and infant ages were also different in the two studies.

As regards SWE combined with the grey-scale ultrasound to diagnose BA in all patients with cholestatic hepatitis, the results showed that the combination of TC sign and AbGB in the parallel test achieved the best diagnostic performance, while grey-scale ultrasound findings in combination with SWE could not increase the AUC and diagnostic accuracy of ultrasound. This indicated that the routine ultrasonography was most reliable in diagnosing BA as also demonstrated by Zhou [26]. A meta-analysis involving 23 studies revealed that the TC sign and the AbGB are the most accurate ultrasonic findings to diagnose BA [33]. This could be explained as follows. First, with the development of ultrasonic imaging technique, the quality of ultrasonic images increases and grey-scale ultrasound can offer sufficient and detailed diagnostic information, especially for the hepatic hilar fibrosis and the detailed structures of gall bladder. Second, Young's modulus value from SWE reflects information regarding the hepatic stiffness. Besides hepatic fibrosis, many other diseases and pathologic factors including inflammation and edema may increase the hepatic stiffness [34], and SWE technique may be affected by many factors, including the detected depth, position, and probe's frequency [35]. Nevertheless, in the serial test, the SWE combined with AbGB achieved the best diagnostic performance, with the AUC being 0.902 and the specificity raising to 100%. It indicated that the combination of SWE and AbGB in the serial test can improve the confidence of radiologists in excluding the diagnosis of BA with ultrasonography.

Hwang SH et al. [24] reported that the TC sign was present in 17% (2/12) of BA infants younger than 30 days and in 56% (34/56) of BA infants older than 30 days. In our study, 14 BA infants resulted without TC sign and 13 of them were younger than 30 days. SWE value in these patients without typical grey-scale ultrasound images is worthy of further investigation. Our study showed that, in patients without TC sign, a cut-off value of hepatic Young's modulus to diagnose BA was 11.55 kPa and the area under the ROC curve was 0.842, with a sensitivity of 78.6% and a specificity of 82.8%. The AbGB was the most useful to diagnose BA and the AUC was 0.960 with a sensitivity of 100% and a specificity of 92.0%. When the SWE was combined with the AbGB in the serial test, the specificity increased from 92.0% to 100%. This indicated that when grey-scale images were atypical, SWE could be used as a useful supplement of gray-scale ultrasound, improving the specificity of BA diagnosis and the confidence of radiologists in excluding BA with ultrasonography.

Although the results of our study were encouraging, there are several limitations. First, our study was a single center one. Thus, a larger multicenter study is needed to further confirm our findings. Second, as the duration between SWE examination and Kasai procedure is a bit long in some patients, we failed to perform correlation analysis between hepatic Young's modulus values and grading of hepatic fibrosis. In the future, we will investigate the correlation between extent of hepatic fibrosis and Young's modulus values.

## 5. Conclusion

In conclusion, both SWE and grey-scale ultrasound have good performance in diagnosing BA. SWE could not only help differentiate BA from cholestatic hepatic diseases but also increase the diagnostic specificity when combined with grey-scale ultrasound in the serial test.

## Figures and Tables

**Table 1:** Gender and age characteristic of the three groups.

Group	all ages			≤30 days old			31-60 days old			61-90 days old		
	n	gender	median	n	gender	median	n	gender	median	n	gender	median
		(M/F)	(range)age		(M/F)	age		(M/F)	age		(M/F)	age
control	62	28/34	35(7-90) days	29	12/17	23 days	20	11/9	48 days	13	5/8	75 days
non-BA	87	58/29	30(5-90) days	48	30/18	20 days	28	21/7	50 days	11	7/4	72 days
BA	51	18/33	43(5-88) days	20	8/12	25 days	19	5/14	49 days	12	5/7	67 days

**Table 2:** Comparison of the Young's modulus values among infants of different genders within the same group and in different groups with same gender.

Group	male		female	
	n	Hepatic Young's modulus values (kPa)	n	Hepatic Young's modulus values (kPa)
control	28	6.69 ± 0.57	34	6.58 ± 0.52
non-BA	58	9.91 ± 2.00^a^	29	9.84 ± 1.49^a^
BA	18	17.94 ± 6.44^ab^	33	17.59 ± 5.65^ab^

^a^ compared with control group, *P*<0.01.

^b^ compared with non-BA group, *P*<0.01.

**Table 3:** Comparison of the Young's modulus values in the same group with different ages and in different groups with same age.

Group	all ages		≤30 days		31-60 days		61-90 days	
	n	Young's modulus	n	Young's modulus	n	Young's modulus	n	Young's modulus
		value (kPa)		value (kPa)		value (kPa)		value (kPa)
Control	62	6.63 ± 0.54	29	6.62 ± 0.55	20	6.69 ± 0.49	13	6.64 ± 0.62
non-BA	87	9.88 ± 1.84^a^	48	9.11 ± 1.43^a^	28	10.75 ± 1.63^a,c^	11	11.04 ± 2.40^a,c^
BA	51	17.72 ± 5.89^a,b^	20	14.34 ± 5.25^a,b^	19	17.82 ± 4.88^a,b,c^	12	23.19 ± 4.21^a,b,c,d^

^a^ compared with control group, *P*<0.01.

^b^ compared with non-BA group, *P*<0.01.

^c^ compared with ≤30 days group, *P*<0.01.

^d^ compared with 31 to 60 days group, *P*<0.01.

**Table 4:** Spearman correlation between Young's modulus values( kPa) and age, TBIL, DBIL, IBIL, and *γ*-GT level in the BA group (n=51).

	mean ± standard deviation (range)	*r*	*P* value
Age (day)	43^a^(5-88)	0.642	<0.001
TBIL (*μ*mol/L)	188.02 ± 68.50(73.71-362.78)	0.467	0.001
DBIL (*μ*mol/L)	119.83 ± 51.44(37.01-245.68)	0.548	<0.001
IBIL (*μ*mol/L)	68.39 ± 24.76(20.20-137.20)	0.222	0.117
*γ*-GT (IU/L)	294.23 ± 168.39(69.00-871.00)	0.678	<0.001

^a^ Data are medians; *r*: correlation coefficient.

**Table 5:** Linear regression analysis of hepatic Young's modulus value ( kPa) correlated with age, TBIL, DBIL and *γ*-GT level.

	Unstandardised coefficients	standard error	standardised coefficients	*t*	*P* value
Constant	5.574	2.125		2.622	0.012
Age (day)	0.099	0.035	0.341	2.842	0.007
TBIL (*μ*mol/L)	0.009	0.029	0.106	0.316	0.753
DBIL (*μ*mol/L)	0.024	0.039	0.209	0.615	0.542
*γ*-GT (IU/L)	0.011	0.004	0.313	2.553	0.014

**Table 6:** Diagnostic performance of hepatic Young's modulus values, typical grey scale ultrasound findings, and their combination to diagnose BA for all infants with cholestatic hepatitis.

	*P* value	AUC	*P *value vs SWE^a^	95%CI	Sensitivity	Specificity	PPV	NPV	Accuracy
SWE	<0.001	0.937		0.894,0.978	84.3%	89.7%	82.7%	90.7%	87.7%
TC	<0.001	0.863	>0.05	0.787,0.939	72.5%	100%	100%	86.1%	89.8%
AbGB	<0.001	0.94	>0.05	0.895,0.986	96.1%	92.0%	87.5%	97.6%	93.5%
SWE or TC	<0.001	0.899	>0.05	0.839,0.959	90.2%	89.7%	83.6%	94.0%	89.8%
SWE or AbGB	<0.001	0.908	>0.05	0.857,0.959	100%	81.6%	76.1%	100%	88.4%
TC or AbGB	<0.001	0.96	>0.05	0.926,0.994	100%	92.0%	87.9%	100%	94.9%
SWE or TC or AbGB	<0.001	0.908	>0.05	0.857,0.959	100%	81.6%	76.1%	100%	88.4%
SWE and TC	<0.001	0.833	<0.05	0.751,0.915	66.7%	100%	100%	83.6%	87.7%
SWE and AbGB	<0.001	0.902	>0.05	0.849,0.975	80.4%	100%	100%	90.6%	93.5%
TC and AbGB	<0.001	0.843	<0.05	0.763,0.923	68.6%	100%	100%	84.5%	88.4%
SWE and TC and AbGB	<0.001	0.814	<0.05	0.728,0.899	62.7%	100%	100%	82.1%	86.2%

^a^ compared with the AUC of SWE; or: in the parallel test; and, in the serial test; PPV: positive predictive value; NPV: negative predictive value.

**Table 7:** Diagnostic performance of SWE, abnormal gallbladder, and their combination to diagnose BA in patients without TC sign.

	*P* value	AUC	*P *value vs SWE^a^	95%CI	Sensitivity	Specificity	PPV	NPV	Accuracy
SWE	<0.001	0.842		0.724, 0.961	78.6%	82.8%	42.3%	96.7%	82.2%
AbGB	<0.001	0.960	<0.05	0.924, 0.996	100%	92.0%	65.0%	100%	93.1%
SWE or AbGB	<0.001	0.874	>0.05	0.806, 0.941	100%	74.7%	37.1%	100%	78.2%
SWE and AbGB	<0.001	0.893	>0.05	0.000, 1.000	78.60%	100%	100%	96.7%	97.0%

^a^ compared with the AUC of SWE; or: in the parallel test; and: in the serial test; PPV: positive predictive value; NPV: negative predictive value.

## Data Availability

The data used to support the findings of this study are available from the corresponding author upon request.
